# The use of Fuller's earth to reduce acetoacetate interference in the measurement of serum creatinine by the Beckman Astra-4 analyser

**DOI:** 10.1155/S1463924682000194

**Published:** 1982

**Authors:** John Thompson

**Affiliations:** Department of Chemical Pathology St. James's University Hospital Beckett Street Leeds LS9 7TF UK


Journal of Automatic Chemistry, Volume 4, Number 2 (April–June 1982), pages 69–71

### The use of Fuller's earth to reduce acetoacetate interference in the measurement of serum creatinine by the Beckman Astra-4 analyser

**John Thompson**

*Department of Chemical Pathology, St. James's University Hospital, Beckett Street, Leeds LS9 7TF, UK*

### Introduction

Several authors have recently re-emphasized the positive interference of acetoacetate in the measurement of creatinine using the Jaffé reaction [1–3]. On theoretical grounds, the measurement of creatinine in the presence of acetoacetate should be possible using a kinetic method [4]. In practice, however, measurements by some instruments employing a kinetic Jaffé method suffer from marked interference by acetoacetate.

Haekel described a procedure for the measurement of creatinine in the presence of acetoacetate, utilizing the adsorption of creatinine onto Fuller's earth, and subsequent simultaneous elution and colour development with alkaline picrate [5 and 6]. One of the variations of his procedure is that it omits a protein precipitation step [5]. In the present work, the procedure omitting a protein precipitation step has been adapted by eluting creatinine from the Fuller's earth and measuring the creatinine in the eluate using a Beckman Astra-4 analyser (Beckman RIIC Ltd, High Wycombe, Buckinghamshire HP12 3NR, UK).

### Materials and methods

### *Apparatus*

Serum creatinine measurements were performed using a Beckman Astra-4 analyser. The instrument was equipped with modules for the measurement of sodium/potassium, bicarbonate, urea, and creatinine, but during this investigation only the sodium/potassium and creatinine modules were programmed for use. The instrument was calibrated using Beckman Astra standard solutions.

### *Reagents*

Acetoacetate was obtained as the lithium salt (Sigma London Chemical Company Ltd, Poole, Dorset BH17 7NH, UK). All other reagents were obtained from B.D.H. Chemicals Ltd (Poole, Dorset BH12 4NN, UK). The Fuller's earth reagent was prepared by adding 6 g of Fuller's earth to 500 ml of deionized water, adding 10 ml of 1·0 ml/l hydrochloric acid, and making the reagent volume up to 1 l with deionized water. Whilst in use, the Fuller's earth was kept in suspension by continuous stirring with a magnetic stirrer. The eluting reagent consisted of 1·0 mol/l potassium hydroxide containing 100 mol/l sodium chloride.

### *Procedure*

200 $\mu$l of serum was added to 5·0 ml of Fuller's earth reagent in a 10 ml glass tube. The tube contents were mixed for 1 min. by gentle inversion and the mixture was then centrifuged for 1 min. at 3000 × g. Using a fine-tipped Pasteur pipette connected to a filter pump, as much as possible of the supernatant liquid was removed without disturbing the Fuller's earth pellet. 400 $\mu$l of eluting reagent was added to the Fuller's earth pellet, and the Fuller's earth resuspended using a vortex mixer. The mixture was gently shaken for 1 min. keeping the tube in a vertical position, then it was centrifuged for 1 min. at 3000 × g and the sodium and creatinine concentrations in the resulting supernatant were measured on the Astra-4. The concentration of creatinine in the original serum sample was calculated as follows:

$$\text{Serum creatinine} = 2 \times \frac{\text{Astra-4 creatinine}}{\text{result}} \times \frac{[\text{Na}]\text{in eluting reagent}}{[\text{Na}]\text{in final supernatant}}.$$

Sodium levels were measured in the eluting reagent and final supernatant. This provides a correction factor to compensate for some liquid being retained by the Fuller's earth pellet after the first centrifugation step.

### Results

### *Stability of creatinine in the eluting reagent*

Since creatinine is unstable under alkaline conditions [7], its stability in the eluting reagent was studied. No change in creatinine concentratiion was found after 1 h incubation in the eluting reagent, but after 2 h the creatinine had fallen to approximately 90% of the original value. All measurements were therefore performed within 1 h of adding the eluting reagent.

### *Comparison of Fuller's earth procedure with the standard Astra procedure*

Creatinine values obtained using the procedure described were compared with values obtained using the standard Astra procedure. The comparison was performed on serum samples referred to the laboratory for electrolyte measurements. Serum samples from patients with ketoacidosis were excluded. The 69 samples used in the comparison consisted of 22 samples with creatinine in the range 50–140 $\mu$mol/l as measured by the standard Astra procedure, 27 samples in the range 140–350 $\mu$mol/l, and 20 samples with serum creatinine in excess of 350 $\mu$mol/l.

Statistical analysis of this data, taking the standard Astra procedure as the *x* axis, and the Fuller's earth procedure as the *y* axis yielded a correlation coefficient of 0·997, a slope of 0·938, and an intercept on the *y* axis of $-10\,\mu$mol/l.

The recovery of endogenous creatinine by the Fuller's earth

0142 0453/82/0402 0069 $05·00 © 1982 Taylor & Francis Ltd procedure was calculated as:

Recovery =

$$\frac{\text{Creatinine result using Fuller's earth procedure}}{\text{Creatinine result using standard Astra procedure}} \times 100\%.$$

The mean recovery was 87% (S.D. = 8%).

### *Recovery of added creatinine*

The recovery of added creatinine was studied by enriching portions of 30 serum samples with creatinine to give an increase in creatinine concentration of 442 μmol/l. The recovery was calculated as:

$$\text{Recovery} = \frac{\text{Creatinine result in enriched sample after Fuller's earth procedure} - \text{Creatinine result in original sample after Fuller's earth procedure}}{442} \times 100\%.$$

The mean recovery was 91% (S.D. = 7%).

### *Effect of acetoacetate in the standard Astra procedure and in the Fuller's earth procedure*

The effectiveness of the Fuller's earth procedure in reducing acetoacetate interference was studied at serum acetoacetate concentrations of 4 mmol/l and 10 mmol/l. Portions of serum were enriched with acetoacetate to give a calculated concentration of 4 mmol/l. The original and enriched sera were then assayed for creatinine using the standard Astra and Fuller's earth procedures. The experiment was then repeated using an added acetoacetate concentration of 10 mmol/l. The results of these two experiments are presented in figure 1 and table 1.

### *Imprecision of the Fuller's earth procedure*

Within-batch between-batch imprecision of the Fuller's earth procedure was determined at three levels of serum creatinine. The results of this study are presented in table 2.

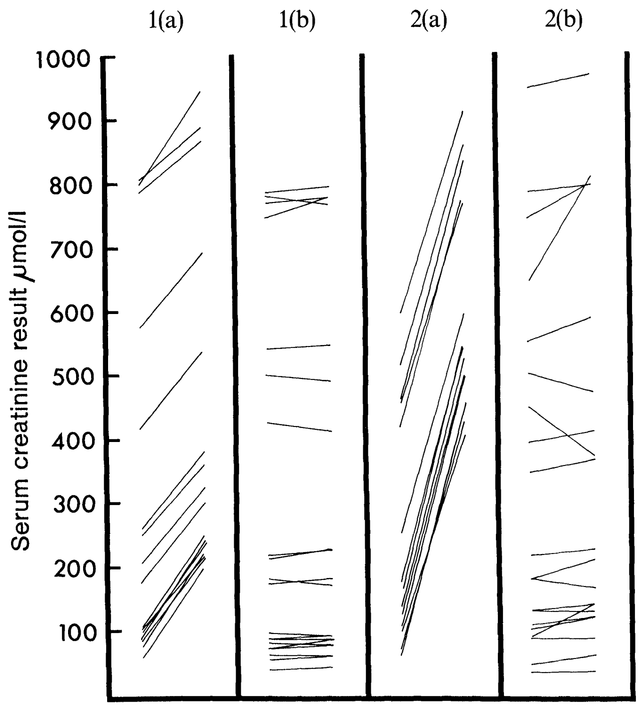

*Figure 1. Effect of acetoacetate on creatinine as measured by the standard Astra and Fuller's earth procedures. 1(a): standard Astra procedure before and after addition of 4 mmol/l acetoacetate; 1(b): Fuller's earth procedure before and after addition of 4 mmol/l acetoacetate; 2(a): standard Astra procedure before and after addition of 10 mmol/l acetoacetate; 2(b): Fuller's earth procedure before and after addition of 10 mmol/l acetoacetate.*

*Table 1. Effect of acetoacetate on creatinine measurement by the standard Astra and Fuller's earth procedures.*

| Analytical procedure | Number of pairs | Added acetoacetate mmol/l | Measured creatinine: Mean pair difference μmol/l | Measured creatinine: Standard deviation μmol/l | Significance of mean pair difference from 0 |
|---|---|---|---|---|---|
| Standard Astra procedure | 20 | 0, 4·0 | 131 | 22 | <0·001 |
| Fuller's earth procedure | 20 | 0, 4·0 | 2 | 11 | 0·650 |
| Standard Astra procedure | 20 | 0, 10·0 | 347 | 32 | <0·001 |
| Fuller's earth procedure | 20 | 0, 10·0 | 12 | 30 | 0·08 |

*Table 2. Precision of creatinine measurement by the Fuller's earth procedure.*

| | Number of observations | Mean creatinine concentration μmol/l | Standard deviation μmol/l | Coefficient of variation % |
|---|---|---|---|---|
| Within-batch precision | 20 | 74 | 6 | 8·2 |
| | 20 | 305 | 10 | 3·4 |
| | 20 | 625 | 13 | 2·0 |
| Between-batch precision | 20 | 41 | 5 | 12·2 |
| | 20 | 242 | 7 | 2·9 |
| | 20 | 494 | 12 | 2·5 |

*Table 3. Laboratory results for a patient in diabetic ketoacidosis.*

| Time | Serum glucose mmol/l | Astra-4 results Sodium mmol/l | Potassium mmol/l | $HCO_3$ mmol/l | Urea mmol/l | Creatinine μmol/l | Fuller's earth procedure creatinine μmol/l |
|---|---|---|---|---|---|---|---|
| 03·00 | 30·4 | 133 | 4·3 | 9 | 8·6 | 279 | 77 |
| 05·00 | 10·4 | 139 | 3·7 | 14 | 7·0 | 205 | 54 |
| 12·00 | 7·1 | 135 | 4·0 | 15 | 6·7 | 108 | 40 |

### Discussion

The Fuller's earth procedure produced somewhat lower values for creatinine than the standard Astra procedure. The lower values could have been due to incomplete recovery of creatinine either at the stage of adsorption of creatinine onto the Fuller's earth, or at the stage of alkaline elution from the Fuller's earth. Alternatively, the lower values could have been due to the removal of some non-creatinine chromogen from serum by the Fuller's earth procedure. The similarity between the observed recovery of endogenous and added creatinine (87% and 91%) suggests that the lower values obtained using the Fuller's earth procedure were mainly due to loss of creatinine at some stage rather than being due to the removal of a positively interfering substance. However, the difference in mean recoveries of endogenous and added creatinine is statistically significant ($p < 0{\cdot}05$), so more than one effect may be operating. Attempts to increase the recovery of creatinine by varying the mixing times of serum and Fuller's earth, and eluting reagent and Fuller's earth were unsuccessful. Varying potassium hydroxide concentration in the eluting reagent also failed to improve the recovery.

Although quantitatively acetoacetate is not the major 'ketone body' in ketoacidosis, it is by far the most important compound in terms of interference in the Jaffé reaction on the Astra-4. Serum acetoacetate levels of 4 mmol/l are not uncommon in ketoacidosis, and it can be seen from figure 1 that the Fuller's earth procedure virtually eliminates the positive interference of acetoacetate at this level. Serum acetoacetate levels of 10 mmol/l represent the extreme and figure 1 shows that acetoacetate interference at this level is not abolished in all the serum samples studied, though the interference is considerably reduced.

The practical effect of acetoacetate interference in creatinine measurement on the Astra-4 is demonstrated in table 3, which shows the results obtained during the treatment of a patient admitted to hospital in diabetic ketoacidosis.

The rapidity with which the Fuller's earth procedure can be performed makes in suitable to emergency investigations. For convenience, the Fuller's earth reagents may be stored in 5 ml aliquots as suggested by Haeckel [5]. It is important to note that the eluate which is analysed on the Astra contains potassium at a concentration of 1·0 mol/l. This causes an increase in potassium in the following sample of 1–2 mmol/l due to carry-over. To combat this it is suggested that the Fuller's earth procedure samples are followed by a sample of deionized water on the Astra sample tray.

### Acknowledgement

The help of Mr A. J. Little of the Department of Chemical Pathology, St. James's University Hospital, in the statistical analysis of the data presented here is gratefully acknowledged.

### References

1. Bowers, L. D., and Wong, E. T., *Clinical Chemistry*, **26** (1980), 55–561.
2. Molitch, M. E., Rodman, E., Hirsch, C. A. and Dubinski, E., *Annals of Internal Medicine*, **93** (1980), 280–281.
3. Glick, M. R., Moorhead, W. R., Oei, T. O. and Moore, G. R., *Clinical Chemistry*, **26** (1980), 1626.
4. Bowers, L. D., *Clinical Chemistry*, **26** (1980), 551–554.
5. Haeckel, R., *Journal of Clinical Chemistry and Clinical Biochemistry*, **18** (1980), 385–394.
6. Haeckel, R., *Clinical Chemistry*, **27** (1981), 179–183.
7. Cannon, R. K. and Shore, A., *Biochemistry Journal*, **22** (1928), 920–929.

### Meeting announcement

*Royal Society of Chemistry: Automated Combination Techniques with Mass Spectrometry*

The Automatic Methods Group and Western Region of the Royal Society of Chemistry will be holding a meeting on 'Automated Combination Techniques with Mass Spectrometry' on Wednesday 12 May 1982. The meeting will be held at the premises of Messrs W. D. & H. O. Wills, Bristol, and the morning session will include a laboratory tour after an introductory paper from T. M. Long of Imperial Tobacco Ltd on the application of automatic techniques to laboratory and process control. The afternoon session will include papers from R. Tanner (Glaxo Group Research) on automated GC/MS assay for Salbutamol in plasma; J. H. Scrivens (ICI Petrochemicals and Plastics Division) on the on-line process control of reactors in chemical plants using MS; C. S. Gutteridge (Cadbury-Schweppes) on the automated identification of micro-organisms using pyrolysis/GC; and from D. E. Games (University College, Cardiff) on automated methods in combined LC/MS.

The registration fee is £7·00 (£4·00 for students). There will be a restriction on the number that can attend the morning session.

*For further information and registration forms please contact Dr C. J. Jackson, HSE Labs., 403 Edgware Road, London NW2 6LN. Tel.: 01 450 8911, ext. 227.*

